# Carotenoids Improve Obesity and Fatty Liver Disease via Gut Microbiota: A Narrative Review

**DOI:** 10.1002/fsn3.70092

**Published:** 2025-03-10

**Authors:** Dorna Hashemi, Mohammad Vahedi Fard, Kimia Mohammadhasani, Mehdi Barati, Elyas Nattagh‐Eshtivani

**Affiliations:** ^1^ Department of Nutrition Sarvestan Branch, Islamic Azad University Sarvestan Iran; ^2^ Department of Nutrition, Food Sciences and Clinical Biochemistry, School of Medicine, Social Determinants of Health Research Center Gonabad University of Medical Sciences Gonabad Iran; ^3^ Department of Pathobiology and Laboratory Sciences North Khorasan University of Medical Sciences Bojnurd Iran

**Keywords:** alcoholic liver disease, carotenoid, gut microbiota, non‐alcoholic fatty liver disease, obesity

## Abstract

Carotenoids are natural micronutrients found in plants and microorganisms, but not synthesized by animals. Carotenoids show various biological activities, including antioxidant properties, regulation of cell growth, and modulation of gene expression and immune responses. The rising global incidence of fatty liver disease (FLD) and obesity highlights the importance of carotenoids in chronic progressive conditions. Gut microbiota (GM) dysbiosis is associated with the development and progression of obesity and FLD due to the effects of metabolites such as lipopolysaccharide (LPS), bile acids (BAs), and short‐chain fatty acids (SCFAs). Furthermore, GM may affect intestinal barrier integrity. This review evaluates the potential impact of carotenoids on GM and intestinal barrier function, and their subsequent effects on obesity and FLD. We searched through a wide range of databases, such as Web of Science, Scopus, EMBASE, and PubMed, to collect data for our non‐systematic review of English literature. Carotenoids such as lycopene, zeaxanthin, fucoxanthin, capsanthin, astaxanthin, and lutein can regulate GM composition and improve obesity and FLD by affecting energy expenditure, food intake, lipid profile, liver fat deposition, liver enzymes, inflammatory markers, glucose homeostasis, and bile acids. These carotenoids improve obesity and FLD through GM metabolites such as SCFAs and LPS. Our findings show that dietary supplementation of lycopene, zeaxanthin, fucoxanthin, capsanthin, astaxanthin, and lutein can positively affect obesity and FLD by regulating GM and intestinal barrier integrity.

## Graphical abstract

Carotenoids are natural micronutrients that are found in plants and microorganisms, but are not synthesized by animals. Our findings showed that dietary supplementation of lycopene, zeaxanthin, fucoxanthin, capsanthin, astaxanthin, and lutein can positively affect obesity and FLD by regulating GM and intestinal barrier integrity.

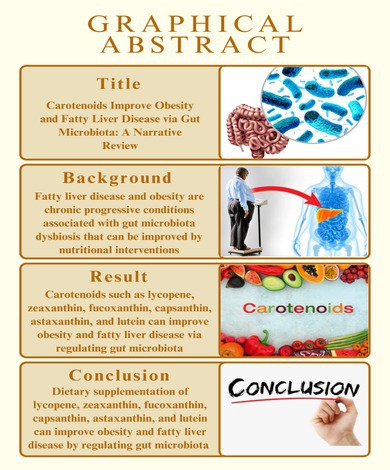

## 1 Introduction

Modernization and changes in lifestyle worldwide have led to numerous diseases and complications (Haththotuwa et al. 2020). Obesity is a global health concern and more than 1 billion people are living with it, in the world (NCD Risk Factor Collaboration (NCD‐RisC) 2024). Also, obesity is increasing alarmingly among children (Balasundaram and Krishna 2024). Obesity increases the risk of diseases, such as diabetes, cardiovascular diseases, and fatty liver disease (FLD) (Cuzmar et al. 2020). FLD is a chronic progressive liver condition marked by the excessive collection of fat in the liver, which is categorized into non‐alcoholic fatty liver disease (NAFLD) and alcoholic liver disease (ALD) (Staufer and Stauber 2023). There is a difference between the pathogenesis of fatty degeneration but inflammatory processes play essential roles in the pathogenesis of FLD. Changes in habits, lifestyle, and culture are the same risk factors for ALD and NAFLD (Degenhardt et al. 2018; Seydel et al. 2017). While NAFLD is closely linked to metabolic disorders like obesity and diabetes, ALD is primarily caused by alcohol abuse disorders (Rehm et al. 2013; Rinella and Charlton 2016). However, factors such as obesity can exacerbate ALD (Inan‐Eroglu et al. 2022). FLD is usually asymptomatic, but some patients may experience symptoms, such as nausea and vomiting, alteration of the sleep–wake cycle, hepatomegaly, abdominal pain, and mood swings (Khoonsari et al. 2017; Patel et al. 2021). ALD and NAFLD are the leading causes of liver cirrhosis, hepatocellular carcinoma (HCC), and death (Younossi et al. 2016). So, obesity and FLD are public health priorities worldwide (Asrani et al. 2019).

A healthy diet is a key factor in controlling obesity and FLD (Ristic‐Medic et al. 2022). Plant‐based dietary patterns, which are a source of phytochemicals as active substances, can prevent and improve obesity and FLD by exerting many biological activities (Ahmadi et al. 2023; Molyneux et al. 2007). These non‐nutritive compounds are found in fruit, vegetables, seeds, whole grains, and various food plant sources (Bahadoran et al. 2015). Also, carotenoids, as a group of phytochemicals, are plant bioactive compounds rich in yellow‐orange fruits and vegetables (Lee et al. 2019). Carotenoids can only be obtained from dietary sources and cannot be synthesized by humans (Böhm et al. 2021). Carotenoids have anti‐inflammatory and antioxidant properties. So, they are known for their beneficial effects (Park et al. 2010; Saini et al. 2015). Several studies have shown that the consumption of dietary carotenoids can attenuate the risk of developing lifestyle diseases, such as obesity and FLD (Lee et al. 2019; Mounien et al. 2019). The composition of gut microbiota (GM) which plays a pivotal role in health status, can be modulated by dietary patterns. So, carotenoids can impact intestinal microbiota through various mechanisms (Eroglu et al. 2023). GM in the large intestine can degrade carotenoids into unknown metabolites like apo‐carotenoids that may have biological effects (Linnewiel‐Hermoni et al. 2014). They can also demonstrate a prebiotic‐like effect. As a result, it leads to positive bacterial changes (Wiese et al. 2019). Some studies indicate that dietary carotenoids can reverse or increase characteristics related to obesity and FLD by beneficial changes in the GM composition and modulating the gut‐liver axis (Eroglu et al. 2023; Rocha et al. 2023).

The intricate correlation between the gastrointestinal tract and the liver, known as the gut‐liver axis, is formed through the portal vein and the mesenteric lymphatic system (Albillos et al. 2020). This demonstrates the significant association between GM and the progression of FLD. Generally, GM comprises six phyla: *Firmicutes*, *Bacteroidetes*, *Verrucomicrobia*, *Fusobacteria*, *Proteobacteria*, and *Actinobacteria*. The predominant phyla are *Firmicutes* and *Bacteroidetes* (Hou et al. 2022). Dysbiosis is defined by a reduction in microbial diversity, an elevation in bacteria such as *Proteobacteria*, and the depletion of beneficial bacteria like *Bacteroides* strains (Humphreys 2020). Therefore, an imbalance in GM can elevate the risk of obesity and liver diseases (Vajro et al. 2013). This study aims to examine the beneficial impact of carotenoids on obesity and FLD by affecting the composition of GM.

## 2 Mechanism Between Gut Microbiota and Fatty Liver Disease

### 2.1 Short‐Chain Fatty Acids

Short‐chain fatty acids (SCFAs) are produced from fiber and non‐digestible carbohydrates by GM (Akhtar et al. 2022). SCFAs have several positive health effects, including energy use and energy intake, improved adiposity, modulation of lipid and glucose metabolism, improved inflammation, and regulation of gut barrier integrity (Nogal et al. 2021; Zhang et al. 2021). SCFAs derived from GM are absorbed into the bloodstream and influence overall body physiology via mechanisms that may involve free fatty acid receptors (Mishra et al. 2020; Stoddart et al. 2008). They are expressed in almost all metabolically active tissues, like adipose tissue and liver, suggesting a potential relationship between GM, SCFAs, and physiological functions (al Mahri et al. 2022; Luo et al. 2021; Mishra et al. 2020). SCFAs are able to elevate the anorectic gut hormones release, including peptide YY and glucagon‐like peptide 1 from enteroendocrine cells and leptin from adipose tissue (Lu et al. 2016). Peptide YY and glucagon‐like peptide 1 inhibit appetite‐stimulating neuropeptide Y (NPY)/agouti‐related peptide (AgRP) neurons while enhancing the activity of appetite‐suppressing pro‐opiomelanocortin/cocaine and amphetamine‐regulated transcript neurons in the hypothalamic arcuate nucleus (Chambers et al. 2015). Leptin elevates the activity of these neurons and inhibits NPY/AgRP neurons as well (Chambers et al. 2015). Therefore, SCFAs modulate appetite and energy intake. SCFA‐activated free fatty acid receptors influence body weight and the capacity for fat storage in adipocytes, along with various functions of adipose tissue, such as adipogenesis, lipolysis, lipid and glucose homeostasis, adipokine release, and the management of low‐grade inflammation (Dewulf et al. 2013; Jiao et al. 2021; Ohira et al. 2013; Xiong et al. 2004). SCFAs activate activated protein kinase (AMPK) that upregulates peroxisome proliferator‐activated receptor α (PPARα), PPARγ, uncoupling protein‐1, fatty acid oxidation, adiponectin, and resistin, leading to reduced adipogenesis, increased browning, and improved glucose and lipid metabolism (May and den Hartigh 2023). Also, AMPK reduces hepatic synthesis of fatty acids by suppressing sterol regulatory element‐binding protein (SREBP)‐1c (Yap et al. 2011). Moreover, SCFAs reduce inflammation and suppress apoptosis of hepatic stellate cells by regulating nuclear factor‐κB (NF‐κB) and toll‐like receptor 4 (TLR4) (Li et al. 2021; May and den Hartigh 2023). Therefore, SCFAs derived from GM improve obesity and liver damage through these mechanisms.

### 2.2 Choline

Choline is an essential nutrient that is important for metabolism and liver function (López‐Sobaler et al. 2020). It is an essential component of membrane phospholipids and is critical for lipid and cholesterol metabolism in the liver. Choline contributes to several physiological pathways and prevents fat deposition in the liver by facilitating fat transportation (Erdman Jr et al. 2012). Furthermore, trimethylamine (TMA) is produced by the metabolization of choline via GM. The liver transforms TMA into trimethylamine‐N‐oxide (TMAO). TMAO contributes to obesity, liver damage, insulin resistance, and inflammation (Barrea et al. 2018; Fennema et al. 2016). Elevated concentrations of TMAO in the bloodstream are regarded as a predictive marker for enhanced production of TMA, thereby reflecting an indirect indicator of modifications in the metabolism of choline and phosphatidylcholine. Inadequate levels of choline can disrupt the synthesis of very low‐density lipoproteins, resulting in the accumulation of triglycerides (TG) and the development of hepatic steatosis (Mehedint and Zeisel 2013).

### 2.3 Intestinal Barrier

The intestinal barrier consists of chemical, microbial, immune, and mechanical barriers that protect against pathogens and toxins invading the lumen (Chen et al. 2021). Increased intestinal permeability is associated with obesity and liver damage by allowing the translocation of pathogens and metabolites, leading to an immune response and inflammation (DiMattia et al. 2023; Liu et al. 2023; Nicoletti et al. 2019). The epithelial layer preserves intestinal barrier permeability (Rios‐Arce et al. 2017). Tight junctions in intestinal epithelial cells are essential for preserving intestinal barrier function and are composed of various junctional molecules, such as zonula occludens (ZO)‐1 and‐2, occludin, and claudin (Lee et al. 2018). The role of tight junctions is regulating paracellular diffusion and detection of intact cell‐to‐cell contacts (Chelakkot et al. 2018; Montalto et al. 2004). GM composition modulates the immune system and regulates the expression of tight junctions through it (Bischoff et al. 2014; Isaacs‐Ten et al. 2020). Also, GM regulates intestinal stem cell function and improves gut barrier integrity and disorders (Ma et al. 2022).

### 2.4 Lipopolysaccharide

Lipopolysaccharides (LPS) are toxins produced by gram‐negative bacteria that affect human health, mainly through immune system interactions (Farhana and Khan 2023). GM dysbiosis and increased intestinal permeability lead to higher LPS invasion into the lumen and bloodstream (Vespasiani‐Gentilucci et al. 2015). LPS binds to lipopolysaccharide‐binding protein (LBP), creating the LBP–LPS complex. This complex is then transferred to either a soluble cluster of differentiation 14 (CD14) or a membrane‐bound CD14. CD14 specifically interacts with TLR4, triggering the activation of the adaptor molecule myeloid differentiation factor 88 (MyD88). MyD88 subsequently activates downstream pathways, such as NF‐κB and mitogen‐activated protein kinase (Wang et al. 2024). Therefore, LPS triggers systemic and local inflammation in the body that enhances liver injury and the degree of obesity by elevating pro‐inflammatory cytokine levels such as interleukin (IL)‐6, IL‐1, and tumor necrosis factor α (TNF‐α) (Cani et al. 2008; Roh and Seki 2013; You et al. 2005).

### 2.5 Bile Acid

Bile acids (BAs) are a large family of molecules that hepatically synthesize cholesterol derivatives (Fleishman and Kumar 2024). BAs are known as metabolic integrators due to their impact on energy expenditure and glucose and lipid metabolism (Chiang and Ferrell 2019). They modulate TG production and energy expenditure by BAs‐farnesoid X receptor and BAs‐Takeda G protein‐coupled receptor 5 (TGR5) respectively (Preidis et al. 2017; Watanabe et al. 2006). Thus, BAs affect the progression of FLD and obesity through these pathways (Shao et al. 2021). Therefore, GM affects fatty liver and obesity by modulating BA profiles (Collins et al. 2023).

## 3 Methods

The primary aim of this narrative review was to investigate “Carotenoids Improve Obesity and Fatty Liver Disease via Gut Microbiota”. A comprehensive search of English‐language literature was conducted using databases such as Web of Science, Scopus, EMBASE, and PubMed. The search was conducted without any time restriction. Studies were searched using the keywords “Obesity”, “Non‐alcoholic Fatty Liver Disease”, “NAFLD”, “Steatohepatitis”, “Fatty Liver, Alcoholic”, “Carotenoid”, “Lycopene”, “Zeaxanthin”, “Fucoxanthin”, “Capsanthin”, “Astaxanthin”, “Lutein”, “Gastrointestinal Microbiome”, “Gut Microbiome”, and “Intestinal Microbiota”. The articles were integrated into our study based on their relevance to the subject matter. Furthermore, extra papers that were familiar to the authors were included.

## 4 Results

### 4.1 Lycopene

Lycopene is a lipophilic carotenoid, rich in vegetables and red fruits like tomatoes, grapefruit, and papaya (Cassileth 2010). Lycopene is an advantageous antioxidant with anti‐inflammatory effects (Imran et al. 2020). Also, the impact of lycopene on GM led to further research into its potential effects on obesity and fatty liver (Table 1).

**TABLE 1:** A review of studies that examined the effect of carotenoids on FLD via gut microbiota.

Authors	Dose and Duration	Population	Effect	Changes in gut microbiota
*Lycopene*				
Tu et al. (2023)	0.1% w/w 19 weeks	ApoE−/− mice	↓ TC, LDL‐C, TNF‐α, MCP‐1, IL‐6 and IL‐1β ↑ HDL‐C ↑ ZO‐1 and occludin expression ↓ serum LPS, D‐LA and DAO	↓ *Actinobacteria* ↓ *Firmicutes*, *Firmicutes*/*Bacteroides* ratio ↑ *Bacteroidetes*, *Verrucomicrobia* ↑ *TM7* ↑ *Akkermansia* and *Alloprevotella*
Wu et al. (2019)	200 mg/kg/day p.o. 12 weeks	C57BL/6 mice	↓ body weight, epididymal adipose tissue weights ↓ blood glucose ↓ TG, TC, LDL‐C, liver TC and TG ↓ TMAO, FMO3 activity ↓ PPARγ, FAS expression in liver ↑ PPARα, CPT‐1 expression in liver	↓ *Actinobacteria* ↑ *TM7* ↓ *Lactobacillus*, *Parabacteroides*, and *Dorea* ↑ *Desulfovibrio*, *Coprococcus*, *Allobaculum*, *Akkermansia*, and *Bifidobacteroides*
Gao et al. (2023)	20 and 60 mg/kg/day p.o. 8 weeks	C57BL/6J mice	↓ body weight gain, WAT ↓ HDL‐C, LDL‐C, TG, TC, insulin, and FBG ↓ LPS ↓ The hepatic steatosis, steatosis scores, inflammation scores, balloning scores, and hepatic TG levels ↓ expression NLRP3, Pro‐Caspase‐1, Caspase‐1, NF‐κB, TLR‐4	↓ *Firmicutes* ↓ *Lachnospiraceae_NK4A136_ group*, *Alistipes*, *Desulfovibrio* ↑ *Alloprevotella*
Pan et al. (2022)	5,10, and 20 mg/kg/day p.o. 30 days	C57BL/6 mice	↑SOD ↓ MDA in small intestine ↑ SIgA, IL‐4, IL‐12, and IFN‐γ secretion in small intestine ↑ length of the intestinal villus and crypt depths of the small intestine ↑ the expression of MyD88, TLR4, TRAF6, p‐P38, TRIF, and p‐NF‐κB p65 in small intestinal ↓ MDA and NO in liver ↑ GSH in liver ↓ IL‐1β, IL‐6, TNF‐α	↓ *Firmicutes*/*Bacteroidota* ratio ↑ *Lactobacillus*, *Ruminococcus*, *Enterorhabdus*, *norank_f__Muribaculaceae*, *Lachnospiraceae_NK4A136_group*, *norank_f__Lachnospiraceae*, and *unclassified_f__Lachnospiraceae* ↓ *Staphylococcus*, *Acinetobacter*, and *Corynebacterium*
Xia et al. (2018)	41.9 g/kg diet tomato powder (containing 2.39 mg Lycopene) 24 weeks	BCO1−/‐BCO2−/− double KO mice	↓The incidence of HCC ↑ hepatic lycopene concentration ↑ hepatic SIRT1 protein and mRNA levels ↑ NAMPT protein and mRNA expression ↑ mRNA expression of the circadian rhythm–related gene expression including Clock, Cry2, Per2, and Wee1 ↓ The average steatosis score ↑ Phosphorylation and total AMPK protein levels ↑ PGC‐1a, ACOX1, CPT1, and PPAR‐a mRNA levels ↓ The expression levels of DGAT1 and CD36 genes ↓ hepatic inflammation foci ↓ hepatic proinflammation biomarkers including NF‐kB p65 acetylation and total protein expression, MCP1, iNOS, TNF‐α, IL‐1β, IL‐6, and IL‐12α mRNA levels	↓ *Bacteroidetes*, *Deferribacteres* and its correspondent genus *Mucispirillum* and species *Mucispirillum schaedleri* ↑ *Firmicutes* ↓ *Bacteroides*, *Clostridium*, and *Parabacteroides* ↑ *Lactobacillus*, *Bifidobacterium*, and *Clostridium sp. Clone‐9* ↓ *Clostridium* and *Clostridium sp.ID4*
Ge et al. (2024)	Lycopene 10 mg/kg, lycopene 5 mg/kg + Nicotinamide mononucleotide 50 mg/kg p.o. 4 weeks	C57BL/6J mice	↓ TNF‐α, IL‐6, and IL‐1β ↑ SOD and GSH‐px activity ↓ MDA content Improved hepatocyte necrosis, inflammatory cell infiltration, and vacuolar degeneration ↓ TLR4 expression ↑ Sirt1 expression ↓ NF‐κB phosphorylation ↑ nuclear factor E2‐related protein 2phosphorylation	↑ *Firmicutes*/*Bacteroidetes* ratio ↑ *Paraprevotella* and *Rikenellaceae RC9 group* ↓ *Bilophila* and *Enterorhabdus* ↑ *Exiguobacterium*, *Kurthia*, *Roseburia*, *Corynebacterium 1*, and *Acinetobacter*
Singh et al. (2016)	Lycopene (5, 10 mg/kg), Lycopene (5 mg/kg) + IMOs (0.5 g/kg), Lycopene (10 mg/kg) + IMOs (1 g/kg) p.o. 12 weeks	Swiss albino mice	↓ weight gain, BMI, and adiposity improved adipose tissue fat mobilization Modulation of Hypothalamic orexigenic and anorectic genes ↑ expression of PPARγ and vWAT ↓ MDA, total nitrite, SOD and GSH ↓ serum TC, TG, LDL‐C, and free fatty acids ↓ liver TG ↑ HDL‐C ↓ insulin and glucagon concentration, insulin resistant, and HOMA2 IR ↓ hyperleptinemia ↓ IL‐6 expression and IL‐1β concentration ↓ NPY and AgRP expression ↓ hepatic GK, PEPCK ↑ GLUT‐4 expression	↑ *Lactobacillus spp*. and *Bifidobacteria*
Huang et al. (2021)	Lyophilized *Momordica cochinchinensis* aril 1 or 3% w/w (containing 0.82 mg/g lycopene) 10 weeks	Wistar rats	↓ body weight gain, food efficiency, epididymis adipose tissues, and adipose cell diameter ↑ daily food intake ↓ plasma TG, TC, LDL‐C ↓ fasting blood glucose, fasting insulin, AUC, HOMA‐IR, plasma leptin, plasma resistin, plasma GIP ↓ liver weight, TG, and cholesterol ↓ plasma GOT and GPT ↑ AMPK phosphorylation and PPAR‐α activity	↓ *Firmicutes*/*Bacteroidetes* ratio
Li et al. (2018)	Tomato powder 41.9 g/kg diet (containing 238.8 mg of lycopene) 24 weeks	BCO1−/‐BCO2−/− double knock‐out mice	↓ severity of hepatic steatosis ↓ hepatic TG ↓ acetylation of FoxO1 ↑ NAMPT expression ↑ AMPK and ACC phosphorylation ↓ DGAT1 expression ↑ PPARα, PPAR‐γ, Adiponectin and adiponectin receptor 2 expression ↓ CD36 transcription ↓ expression of TNF‐α, IL‐6, and IL‐1	↓ *Clostridium* ↓ *Clostridium sp. ID4*, *Clostridium disporicum*
Wiese et al. (2019)	7 and 10 mg/day p.o. 30 days	Obese men and women	↓ LDL‐px and IOD ↓ serum LDL‐C and TG ↑ Lipoprotein O_2_ and StO_2_	↑ *Actinobacteria* ↑ *Bifidobacterium adolescentis* and *Bifidobacterium longum*
*Zeaxanthin*				
Xie et al. (2021)	20 mg/kg/day p.o. 22 weeks	C57BL/6N mice	↓ body weight, inguinal WAT, epididymal WAT, mesentric WAT, and perirenal WAT ↓ liver lipid deposition and liver weight ↓ TG, TC, LDL‐C, and free fatty acids ↑ HDL‐C ↓ GPT and GOT ↓ leptin, ↑irisin ↓ GTT AUC and ITT AUC	↑ *Firmicutes* ↓ *Proteobacteria* ↑ *Clostridia* ↓ *Desulfovibrio*
*Fucoxanthin*				
Sun et al. (2020)	0.05% and 0.1% of diet 4 weeks	C57BL/6J mice	↓ body weight gain ↓ mean adiposity size and WAT index ↓ fasting blood glucose, insulin, HOMA‐IR ↑ HDL‐C ↓ liver weight, steatohepatitis scores ↓ serum LPS ↓ ileum IL‐6, TNF‐α levels and ↑ IL‐10 ↓ IL‐6 and TNF‐α and ↑ IL‐10 mRNA expression	↓ *Firmicutes* ↑ *Bacteroidetes* ↑ *Bacteroidales_S24‐7_group*, *Enterococcaceae*, *Bifidobacteriaceae*, *Ruminococcaceae* ↑ *Anaerotruncus*, *Lactobacillus_equicursoris*, *Romboutsia*, *Lactococcus_lactis*, *Enterococcus_durans*, *Lactobacillus_helveticus*, *Lactobacillus_gasseri*, and *Lactococcus_raffinolactis*, *Streptococcus* ↓ *Faecalibaculum*, *Lachnoclostridium* and *Lachnospiraceae_NK4A136_group*
Hao et al. (2024)	80, 160, and 320 mg/kg/day p.o. 12 weeks	C57BL/6J mice	↓ body weight gain, feed efficiency, WAT, liver weight ↓ AUC ↓ TG, LDL‐C, ALT, TNF‐α and IL‐6 ↓ lipid deposition in liver ↑ total bile acid ↑ TGR5 and farnesoid X receptor expression ↓ FGF15 expression	*Lachnospiraceae* and *Oscillospiraceae*. *o_Lachnospirales*, *c_Clostridia, f_Lachnospiraceae*, *o_Oscillospirales*, *g_Lachnospiraceae_NK4A136_group*, *f_Oscillospiraceae*, and *g_norank_f_Oscillospiraceae* were predominant
Zhou et al. (2024)	35 and 60 mg/kg/day p.o. 8 weeks	C57BL/6J mice	↓ body weight gain, energy intake, white adipose tissue ↓ blood glucose ↓ TG, TC, AST, liver TG, liver weight ↑ HDL‐C ↓ Lipids and lipid‐like molecules ↑ ursocholanic acid ↓ ursodeoxycholic acid	↑ *Lachnospiraceae_NK4A136_group*, *Desulfovibrio*, *Blautia*, and *Lachnospiraceae_UCG‐006*
Guo et al. (2019)	10 and 50 mg/kg/day *Nitzschia laevis* (dominant pigment: Fucoxanthin) p.o. 8 weeks	C57BL/6J mice	↓ body weight gain and WAT ↑ expression of PGC1, UCP1, and SREBP1C ↓ liver weight ↑ Occludin expression	Not significant
*Capsanthin*				
Wu et al. (2019)	200 mg/kg/day p.o. 12 weeks	C57BL/6J mice	↓ body weight gain ↓ blood glucose ↓ TG, TC, and LDL‐C ↓ TMAO	↓ *Firmicutes* ↑ *Bacteroidetes* ↑ *Bifidobacterium*, *Allobaculum*, *Coprobacillus*, *Akkermansia*, and *Blautia* ↓ *Bilophila*, *Helicobacter*, *Lactobacillus*, *[Prevotella], Parabacteroides*, *Bacteroides*, *Odoribacter*, and *Ruminococcus*
*Asthaxanthin*				
Wang et al. (2019)	Astaxanthin (0.005%) and (0.01%), X. dendrorhous (10% w/w), X. dendrorhous (20% w/w) 8 weeks	C57BL/6 mice	↓ body weight gain and body fat index ↓ Plasma TG and cholesterol ↓ Liver TG and cholesterol	↓ *Firmicutes*/*Bacteroidetes* ratio ↓ *Proteobacteria* ↑ *Verrucomicrobia* ↑ *Akkermansia* and *Bacteroides*
Wang et al. (2021)	5, 50, and 100 mg/kg/day p,o. 3 weeks	C57BL/6 mice	↓ body weight gain, liver weight, and food efficiency ratio ↓ perirenal and epididymal adipose tissue ↓ Serum TC, TG and LDL‐C ↑ serum HDL‐C ↓ hepatic TG, TC ↑ Adiponectin receptor 1, PGC‐1α, PPARα, AMPK and liver X receptor α expression ↓ SREBP1c, FAS and SCD‐1	↓ *Firmicutes*, *Proteobacteria* ↑ *Bacteroidetes*, *Actinobacteria* ↑ *S24‐7*, *Bacteroidales*, *Ruminococcaceae*, and *Akkermansia* ↓ *Bifidobacterium*
Liu et al. (2018)	50 mg/kg/day p.o. 12 weeks	C57BL/6J mice	↓ Liver fat accumulation, Hepatic steatosis ↓ TG, LDL‐C ↓ ALT, AST ↓ TNF‐α, IL‐1, IL‐6, macrophage inflammatory protein −2	↓ *Bacteroidetes* and *Proteobacteria* ↑ *Verrucomicrobia* ↓ *Butyricimonas*, *Bilophila*, and *Parabacteroides* ↑ *Akkermansia*
Li et al. (2022)	60 mg/kg/day p.o. 10 weeks	ICR mice	↓ body weight gain, daily food intake, lipid droplet vacuoles, liver index, and epididymal fat index ↓ serum and liver TG, TC, LDL‐C ↑ serum and liver HDL‐C, ALT, and AST ↑ SOD, catalase, FRAP ↓ MDA ↓ IL‐17A, SREBP‐1, ACC, and FAS expression ↑ p‐AMPK, nuclear factor E2‐related protein 2, and heme oxygenase‐1 expression	↓ *Desulfovibrionaceae* ↑ *Lactobacillus* ↑ *[Eubacterium]_xylanophilum_group*, *Candidatus_Saccharimonas*, *Lactocucus*, *Faecalibacterium*, *Dubosiella* ↓ *Rikenella*
Wang et al. (2022)	0.25%, 0.5%, and 0.75% of diet 9 weeks	C57BL/6 mice	↓ body weight gain, energy intake ↓ liver weight, liver visual adipose tissue, liver TG and TC ↓ serum TG, TC, LDL‐C ↓ Production of fatty droplets and apoptosis rate in liver ↓ serum AST, steatohepatitis score ↑ antioxidant capacity, catalase, SOD, and GSH ↓ expression of ACC, FAS, and SCD‐1 ↑ expression of CPT‐1, LXRa, CYP7A1, and CYP27A1	↓ *Firmicutes* and *Proteobacteria* ↑ *Akkermansia* and *Parabacteroides*
Zhang et al. (2024)	100 mg/kg/day p.o. 10 weeks	Kunming mice	↓ serum ALT, AST, and TG ↓ TNF‐α, IL‐6, IL‐1β serum and hepatic levels ↑ hepatic levels of SOD, GSH‐PX, antioxidant capacity ↓ MDA hepatic levels ↑ fecal SCFAs, acetic acid, isobutyric acid, valeric acid, propionic acid ↓ LPS	↓ *Firmicutes*, *Firmicutes*/ *Bacteroidetes* ratio ↑ *Bacteroidetes* ↓ *Lactobacillus* ↑ *norank_f__norank_o_Clostridia_UCG‐014*, *norank_f__Lachnospiraceae*, *Prevotellaceae_UCG‐001*, *unclassified_f_Lachnospiraceae*, *unclassified_f_Prevotellaceae*
Ren et al. (2023)	60 mg/kg/day, 60 mg/kg/day astaxanthin +30 mg/kg/day sorafenib 18 days	BALB/C mice	↑ quantity of tumor‐associated macrophages, infiltration of CD8+ T ↑ Tumor Granzyme B and IL‐2 ↑ serum TNF‐α and INF‐γ ↑ genetic transcription CXCL9, CXCL10, CXCR3 ↑ mRNA expression of Cluster of differentiation 40	↑ Akkermansia and Faecalibaculum ↓ Anaerotrunces Gemella, Colidextribacter, Staphylococcus, and Enterococcus
*Lutein*				
Zhao et al. (2023)	12, 24, 48 mg/kg/day p.o. 14 weeks	Wistar rats	↓ Liver pathology scores ↓ serum ALT, AST, and TG ↑ expression of ADH1, ALDH2, heme oxygenase‐1, and IκB‐α ↓ expression of NF‐κB, TLR4, MyD88, and CYP2E1 ↑ GSH‐Px, SOD, catalase, and antioxidant capacity in liver ↓ TNF‐α. IL‐1β, and LPS levels ↑ ileum length, villus length, villus length/crypt depth ratio ↓ intestinal villi breakage and atrophy, FABP2 levels ↑ expression of Claudin‐1, Occludin and ZO‐1	↑ *Eubacterium ruminantium* , *Faecalibacterium*, *Subdoligranulum*, *Coprobacter*

Tu et al. (2023) in their animal study assessed the effect of lycopene intake on high‐fat diet (HFD)‐fed mice. They found that lycopene intake decreased total cholesterol (TC), low‐density lipoprotein cholesterol (LDL‐C), monocyte chemoattractant protein‐1 (MCP‐1), TNF‐α, IL‐6, and IL‐1β while increasing high‐density lipoprotein cholesterol (HDL‐C). Lycopene improved gut barrier integrity by elevating ZO‐1 and occludin expression. It also reduced serum LPS, d‐lactate (D‐LA), and d‐amino acid oxidase (DAO) concentrations. In terms of GM, lycopene attenuated the abundance of *Actinobacteria*, *Firmicutes*, and the *Firmicutes*/*Bacteroides* ratio while elevating the abundance of *Bacteroidetes*, *Verrucomicrobia*, *TM7*, *Akkermansia*, and *Alloprevotella*. *Firmicutes* were negatively related to HDL‐C while positively associated with serum TC, LDL‐C, D‐LA, DAO, IL‐6, MCP‐1, and IL‐1β levels. The *Firmicutes*/*Bacteroides* ratio was associated with serum TC, LDL‐C, LPS, MCP‐1, D‐LA, and IL‐6 positively, while serum HDL‐C was related to it negatively. *Bacteroides* were positively correlated with serum HDL‐C while negatively related to LDL‐C, TC, MCP‐1, IL‐6, DAO, LPS, and D‐LA. All positive and negative associations were the same between *Bacteroides* and *Verrucomicrobia* except for serum TC levels. Furthermore, *Alloprevotella* and *Akkermansia* had a positive association with serum HDL‐C while showing a negative association with serum IL‐6, IL‐1β, TNF‐α, TC, LDL‐C, MCP‐1, DAO, and D‐LA.

Wu et al. (2019) evaluated the effect of lycopene supplementation on HFD‐fed mice. There was a significant association between lycopene consumption and lower body weight, epididymal adipose tissue weights, blood glucose, LDL‐C, TG, TC, liver TC, TG, TMAO, flavin‐containing monooxygenase 3 (FMO3) activity, and PPARγ and fatty acid synthase (FAS) expression in the liver. Furthermore, it reduced the expression of carnitine palmitoyl transferase‐1 (CPT‐1) and PPARα in the liver. Lycopene changed the GM composition and attenuated the abundance of *Actinobacteria*, *Lactobacillus*, *Parabacteroides*, and *Dorea*, whereas increasing the abundance of *TM7*, *Desulfovibrio*, *Coprococcus*, *Allobaculum*, *Akkermansia*, and *Bifidobacteroides*.

**FIGURE 1:**
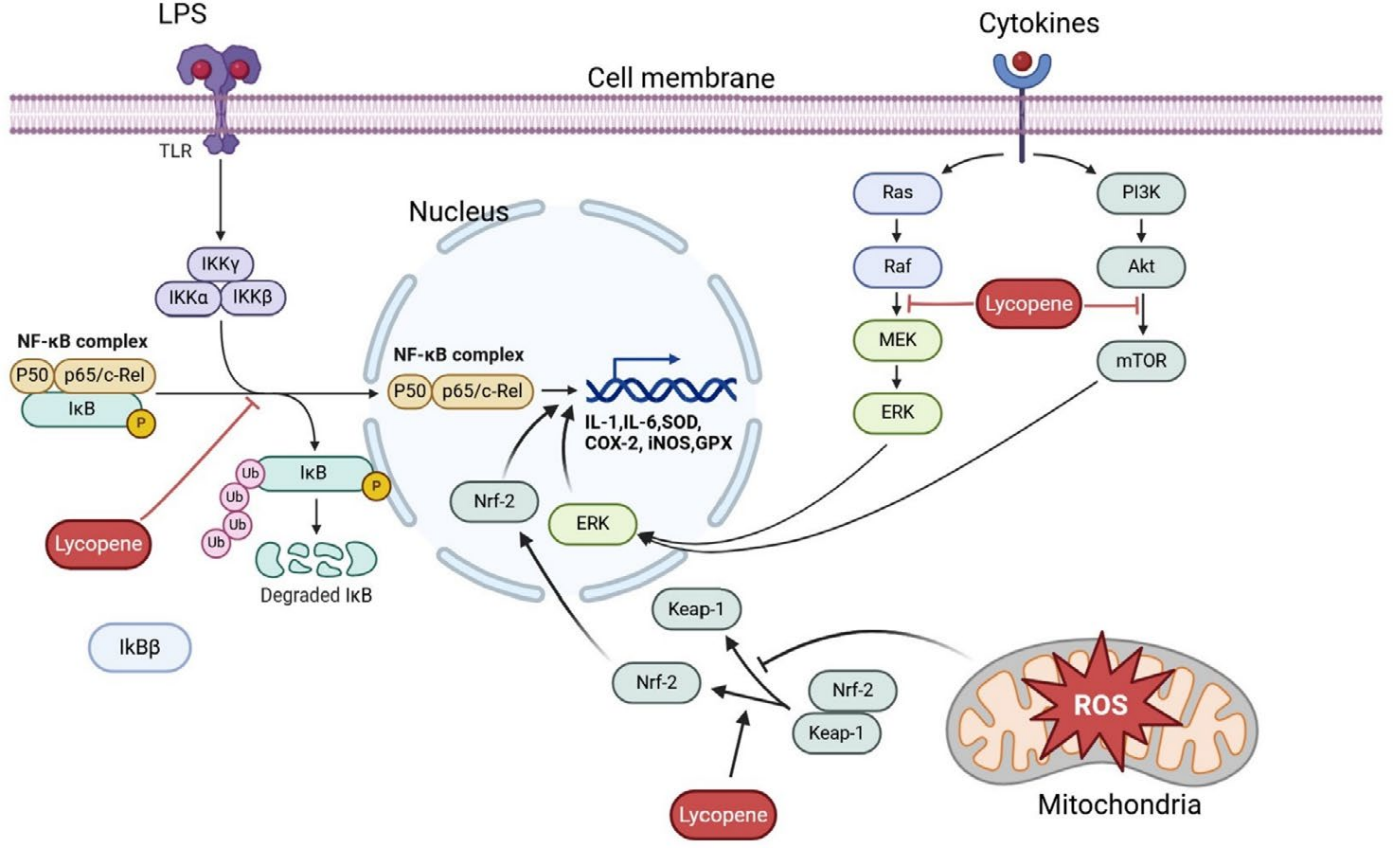
Lycopene reduces the expression of inflammatory mediators such as IL‐6, IL‐1, and TNF‐α in small and large intestinal cells. It also reduces the oxidase damage by enhancing the expression of anti‐inflammatory mediators such as SODs and GSH enzymes. IL‐1; Interleukin‐1, Interleukin 6, TNF‐α; Tumor necrosis factor, LPS; Lipopolysaccharide, SOD; Superoxide dismutase, ROS; reactive oxygen species, COX‐2; Cyclooxygenase‐2, iNOS; Inducible nitric oxide synthase, GPX; Glutathione peroxidase, TLR; toll‐like receptor, Nrf‐2; nuclear factor erythroid 2, Mek; mitogen‐activated protein kinase, ERK; extracellular signal‐regulated kinase, PI3K; phosphatidylinositol‐3‐kinase, Akt; protein kinase B, mTOR; mammalian target of rapamycin, Keap‐1; Kelch‐like ECH‐associated protein 1.

Gao et al. (2023) studied mice fed a high‐fat and high‐fructose diet. They found that mice in the low‐dose and high‐dose lycopene groups had reduced weights of white adipose tissue, body weight gain, HDL‐C serum concentrations, LPS, LDL‐C, alanine transaminase (ALT), hepatic TG, and IL‐6 levels. Furthermore, they found that lycopene may have a preventive effect on NAFLD by inhibiting nucleotide‐binding domain, leucine‐rich–containing family, pyrin domain–containing‐3 (NLRP3), Pro‐Caspase‐1, Caspase‐1, NF‐κB, and TLR‐4 expression. In terms of GM, lycopene reduced the abundance of destructive bacteria, like *Firmicutes*, *Lachnospiraceae_NK4A136_group*, *Alistipes*, and *Desulfovibrio*, and increased SCFAs‐producing bacteria *Allobaculum* and attenuated GM dysbiosis. They recognized that there was a positive relationship between fecal levels of *Lachnospiraceae_NK4A136_group* and serum lipids, LPS, IL‐6, liver TG, as well as mRNA levels of NF‐κB and TLR4. Also, *Alloprevotella* had a negative association with serum lipids, insulin, fasting blood glucose, homeostatic model assessment for insulin resistance (HOMA‐IR), liver TG, as well as mRNA levels of NLRP3, NF‐κB, and Caspase‐1. *Alistipes* was significantly related to mRNA levels of TLR‐4 in the liver Figure 1.

In another study, Pan et al. (2022) used C57BL/6 mice to show the effect of lycopene on NAFLD. They found a significant relationship between lycopene and lower concentrations of liver IL‐6, IL‐1β, TNF‐α, malondialdehyde (MDA), and nitric oxide. Also, lycopene increased liver superoxide dismutase (SOD) and glutathione (GSH), upregulated the expression of key proteins in the small intestine, increased the length of the intestinal villus, crypt depths of the small intestine, and secretion of secretory immunoglobulin A (SIgA), interferon‐γ, IL‐12, and IL‐4 in the small intestine. Lycopene regulated GM by reducing the *Firmicutes*/*Bacteroidota* ratio and reducing the abundance of *Staphylococcus*, *Acinetobacter*, and *Corynebacterium*. Furthermore, it increased the relative abundance of *Lactobacillus*, *Ruminococcus*, *norank_f__Lachnospiraceae*, *unclassified_f__Lachnospiraceae*, *Enterorhabdus, norank_f__Muribaculaceae*, and *Lachnospiraceae_NK4A136_group*.

Xia et al. (2018) found that tomato powder feeding inhibited hepatic tumorigenesis and reduced the incidence of HCC. Also, it has the potential to enhance silent information regulator sirtuin 1 (SIRT1) activity and nicotinamide phosphoribosyltransferase (NAMPT) expression, as well as regulate the expression of genes related to circadian rhythms, which control the inflammation related to obesity and the development of cancer associated with metabolic syndrome. Tomato powder feeding modulated lipid metabolism, inhibited the progression of hepatic steatosis, and reduced the expression of proinflammatory‐related genes. In terms of GM, tomato powder feeding changed the microbial diversity. The abundance of *Bacteroidetes* and *Deferribacteres* was reduced by tomato powder feeding, but the abundance of *Firmicutes* was increased.

In another study, Ge et al. (2024) evaluated the effect of lycopene on acute liver injury among C57BL/6J mice. There was a significant correlation between lycopene and improved hepatocyte necrosis, vacuolar degeneration, inflammatory cell infiltration, and modulated expression of genes associated with oxidative stress and inflammatory pathways. Lycopene also reduced TNF‐α, IL‐6, IL‐1β, and MDA content while elevating the activity of SOD and GSH‐px. In terms of GM, it increased the abundance of *Paraprevotella*, *Rikenellaceae RC9 group*, *Firmicutes*/*Bacteroidetes* ratio, *Kurthia*, *Exiguobacterium*, *Roseburia*, *Corynebacterium 1*, and *Acinetobacter*. Furthermore, lycopene reduced the abundance of *Bilophila* and *Enterorhabdus*.

Singh et al. (2016) studied HFD‐fed mice that induce systemic inflammation and disrupt gut microbial composition. It can also lead to NAFLD and liver inflammation. They found that a symbiotic combination of Isomalto‐oligosaccharides (a prebiotic) and lycopene reduces serum inflammatory cytokines, insulin sensitivity, glucose tolerance, weight gain, adiposity, and liver inflammation. They also showed that lycopene can decrease IL‐6 expression, NPY and AgRP expression, and elevate glucose transporter type 4 (GLIUT‐4) expression. In terms of GM, it elevated the *Enterobacteriaceae* population and prevented the decrease in *Lactobacillus spp*. and *Bifidobacteria*. Furthermore, lycopene increased individual SCFA concentrations and improved gut barrier integrity.

Huang et al. (2021) used Wistar rats to indicate the effect of lycopene on NAFLD. They found that lycopene intake reduced body weight gain, food efficiency, epididymis adipose tissues, adipose cell diameter, plasma TG, plasma TC, plasma LDL‐C, fasting blood glucose, fasting insulin, area under the curve (AUC), HOMA‐IR, plasma leptin, plasma resistin, plasma glucose‐dependent insulinotropic polypeptide (GIP), liver weight, liver TG, liver cholesterol, plasma glutamate pyruvate transaminase (GPT) and oxaloacetate transaminase (GOT) while increasing daily food intake, AMPK phosphorylation, and PPAR‐α activity. It changed the composition of GM by attenuating the Firmicutes/Bacteroidetes ratio.

Using HFD‐induced NAFLD mice, Li et al. (2018) showed a significant association between lycopene intake and reduced severity of hepatic steatosis, liver TG, expression of TNF‐α, IL‐6, and IL‐1. It also decreased the transcription of CD36 (liver fatty acid transporter), acetylation of FoxO1(regulator of glycogenolysis and gluconeogenesis), and expression of diacylglycerol‐acyltransferase 1 (DGAT1) while increasing the expression of PPARα, PPAR‐γ, adiponectin receptor 2, adiponectin, and NAMPT. In terms of GM, it reduced the abundance of *Clostridium*, *Clostridium sp. ID4*, and *Clostridium disporicum* .

Wiese et al. (2019) found the effect of lycopene on GM, blood, and liver metabolism of 15 men and 15 women with moderate obesity. By the end of their trial, changes in the GM profile were observed in all lycopene groups, with an increased abundance of *Bifidobacterium adolescentis* and *Bifidobacterium longum* . Furthermore, they recognize that lycopene was able to decrease inflammatory oxidative damage (IOD) and LDL‐Px.

According to the studies, we discovered that the consumption of lycopene affects the GM and intestinal barrier function with a positive effect on obesity and fatty liver. Further studies, especially human clinical trials, are needed to confirm these findings.

### 4.2 Zeaxanthin

Zeaxanthin is a type of carotenoid pigment found in peppers, carrots, berries, and corn. It also has animal food sources such as egg yolks and certain kinds of fish, like trout and salmon (Abdel‐Aal et al. 2013). As an antioxidant, zeaxanthin has eye‐protective and anti‐inflammatory activities and modulates lipid metabolism (Mrowicka et al. 2022; Tuzcu et al. 2017). Recently, zeaxanthin's effects on obesity and chronic diseases have been considered (Table 1).

Xie et al. (2021) examined the effect of zeaxanthin supplementation on HFD‐fed C57BL/6N mice. They found that zeaxanthin intake reduced body weight, inguinal white adipose tissue (WAT), epididymal WAT, perirenal WAT, mesenteric WAT, liver lipid deposition, liver weight, TG, TC, LDL‐C, free fatty acids, GPT, GOT, AUC, and leptin. Furthermore, it increased HDL‐C and irisin levels and increased the expression of genes correlated with thermogenesis. In terms of GM, zeaxanthin elevated the abundance of *Firmicutes* and *Clostridia*, whereas it reduced the abundance of *Proteobacteria* and *Desulfovibrio*. *Firmicutes* and *Clostridia* were positively associated with the expression of genes correlated with thermogenesis in the inguinal WAT and irisin and HDL‐C. *Desulfovibrio* and *Proteobacteria* were positively associated with body weight, inguinal WAT weight, and serum TG and TC levels.

The previous study showed that zeaxanthin has potential to improve obesity and even fatty liver via modulating GM composition, but more animal and human studies are needed before drawing conclusions.

### 4.3 Fucoxanthin

Fucoxanthin is a xanthophyll carotenoid with beneficial health effects (Bae et al. 2020). It is rich in brown seaweed and shows antioxidant, anti‐inflammatory, and anti‐hyperlipidemic properties (Kim and Pangestuti 2011). Fucoxanthin can modulate GM composition and modulate metabolic functions via GM (Guo et al. 2024). Recently, the effects of fucoxanthin on chronic diseases and obesity via GM modulation have been considered in several studies (Table 1).

**FIGURE 2:**
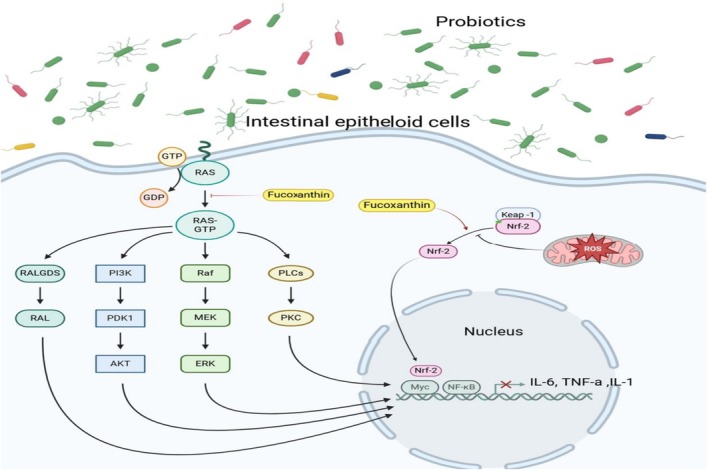
Fucoxanthin increased the abundance of Bacteroidetes, Bacteroidales_S24‐7_group, Enterococcaceae, Bifidobacteriaceae, Ruminococcaceae, *Anaerotruncus*, *Streptococcus*, *Lactobacillus_equicursoris*, *Romboutsia*, *Enterococcus_durans*, *Lactococcus_lactis*, *Lactobacillus_helveticus*, *Lactobacillus_gasseri*, and *Lactococcus_raffinolactis* while decreasing the abundance of Faecalibaculum, Lachnoclostridium, and Lachnospiraceae_NK4A136_group. It suppresses inflammation signals in epithelioid cells; furthermore, it suppresses the expression of IL‐6, IL‐1, and TNF‐a in these cells of the large intestine. IL‐1; Interleukin‐1, Interleukin 6, TNF‐a; Tumor necrosis factor, ERK; extracellular signal‐regulated kinase, PI3K; phosphatidylinositol‐3‐kinase, Akt; protein kinase B, Keap‐1; Kelch‐like ECH‐associated protein 1, Nrf‐2; nuclear factor erythroid 2.

Sun et al. (2020) examined the effect of fucoxanthin intake on obesity among HFD‐fed mice. They found that fucoxanthin supplementation reduced body weight gain, mean adiposity size, WAT, insulin, fasting blood glucose, HOMA‐IR, liver weight, steatohepatitis scores, serum LPS, ileum IL‐6, ileum TNF‐α, mRNA expression of TNF‐α and IL‐6. Also, it increased HDL‐C, ileum IL‐10, and mRNA expression of IL‐10. In terms of GM, fucoxanthin elevated the abundance of *Bacteroidetes*, *Bacteroidales_S24‐7_group*, *Enterococcaceae*, *Bifidobacteriaceae*, *Ruminococcaceae*, *Anaerotruncus*, *Streptococcus*, *Lactobacillus_equicursoris*, *Romboutsia*, *Lactococcus_lactis*, *Enterococcus_durans*, *Lactobacillus_helveticus*, *Lactobacillus_gasseri*, and *Lactococcus_raffinolactis*, whereas it attenuated the abundance of *Lachnospiraceae_NK4A136_group, Faecalibaculum*, and *Lachnoclostridium*. *Faecalibaculum* was associated with body weight gain, WAT index, steatohepatitis scores, adipocyte size, liver weight, LPS, HOMA‐IR, serum glucose, and TNF‐α positively while negatively associated with IL‐10 levels. *Lachnospiraceae_NK4A136_group* and *Lachnoclostridium* were positively correlated with fasting blood glucose, adipocyte size, HOMA‐IR, and mRNA expression of TNF‐α. Furthermore, *Lachnospiraceae_NK4A136_group* was negatively related to IL‐10 levels. On the other hand, *Lactobacillus* and *Bifidobacterium* were associated with adipocyte size, body weight gain, LPS, liver weight, HOMA‐IR, or serum glucose negatively Figure 2.

Hao et al. (2024) evaluated the effect of fucoxanthin supplementation on HFD‐fed mice. They observed a significant relationship between fucoxanthin consumption and reduced body weight gain, feed efficiency, WAT, liver weight, AUC, ALT, LDL‐C, TG, IL‐6, TNF‐α, and fibroblast growth factor 15 (FGF15) expression. On the other hand, it increased total bile acid, and TGR5 and farnesoid X receptor expression. Fucoxanthin intake changed GM composition; *Lachnospiraceae* and *Oscillospiraceae* were predominant in the group of mice that consumed fucoxanthin. Also, *c_Clostridia*, *g_Lachnospiraceae_NK4A136_group*, *f_Lachnospiraceae*, *o_Oscillospirales*, *f_Oscillospiraceae*, *o_Lachnospirales*, and *g_norank_f_Oscillospiraceae* were predominant, too.

In another study, Zhou et al. (2024) assessed the effect of fucoxanthin on obesity among 36 mice. There was a significant correlation between fucoxanthin intake and lower body weight gain, energy intake, WAT, lipids and lipid‐like molecules, blood glucose, TG, TC, aspartate aminotransferase (AST), liver TG, and liver weight while increased HDL‐C. Moreover, it reduced the abundance of ursodeoxycholic acid and increased ursocholanic acid. The GM was regulated, and fucoxanthin increased the abundance of *Desulfovibrio*, *Lachnospiraceae_UCG‐006*, *Lachnospiraceae_NK4A136_group*, and *Blautia*.

Guo et al. (2019) examined the effect of fucoxanthin on obesity. They found that fucoxanthin intake reduces body weight gain, WAT, and liver weight, whereas it elevates peroxisome proliferator‐activated receptor‐gamma coactivator 1 (PGC1), mitochondrial uncoupling protein 1 (UCP1), SREBP1C, and Occludin expression. Fucoxanthin enriched the abundance of some beneficial GM bacteria and reduced the abundance of harmful bacteria, but they were not significant. A lower dose of fucoxanthin in *Nitzschia laevis* might be the reason for insignificant changes in GM.

Based on the previous studies, we found that fucoxanthin intake may improve obesity and fatty liver via regulation of GM composition. More animal and human studies are necessary to find the exact effect of fucoxanthin on GM and have a positive effect on fatty liver and obesity.

### 4.4 Capsanthin

Capsanthin is an orange‐red colored pigment usually found in *Capsicum annuum* species of plants, *Asparagus officinalis* , Lilium, and red‐colored fruits and vegetables (K. Patel and Patel 2024). Capsanthin has shown antioxidant, antihyperlipidaemic, anti‐adipogenic, and cardioprotective activities (Kennedy et al. 2021). Thus, further research is needed to explore its potential effect on obesity (Table 1).

Wu, Gao, et al. (2020) studied the effect of capsanthin on 48 HFD‐fed C57BL/6J mice. There was a significant relationship between capsanthin supplementation and lower body weight gain, blood glucose, TG, LDL‐C, TC, and TMAO. However, daily food intake was not reduced significantly. In terms of GM, capsanthin decreased the abundance of *Firmicutes* and increased the abundance of *Bacteroidetes* at the phylum level. Also, it reduced the abundance of *Bilophila*, *Helicobacter*, *Lactobacillus*, *[Prevotella]*, *Parabacteroides*, *Bacteroides*, *Odoribacter*, and *Ruminococcus* while attenuating the abundance of *Bifidobacterium*, *Allobaculum*, *Coprobacillus*, *Blautia*, and *Akkermansia*.

The previous study showed that capsanthin may have the potential to improve obesity by influencing the composition of GM. However, further studies involving both animals and humans are necessary before concluding.

### 4.5 Astaxanthin

Astaxanthin is a lipid‐soluble xanthophyll carotenoid with antioxidative properties and notable efficacy in preventing and treating conditions like cancer and diabetes (Guerin et al. 2003). It is found widely in nature, such as shellfish, crustaceans, and various plant sources (Xu et al. 2019). It has received approval for its inclusion in food products as a dietary supplement, colorant, and antioxidant, and it has become a high‐demand product (Kim et al. 2022). Astaxanthin has the potential to modulate GM composition and improve metabolic functions (Wu, Lyu, et al. 2020). Recently, the effect of astaxanthin on obesity and fatty liver has been considered in several studies (Table 1).

Wang et al. (2019) evaluated the effect of astaxanthin on obesity among 40 HFD‐fed C57BL/6 mice. They found that astaxanthin supplementation significantly attenuated body fat index, body weight gain, plasma TG, plasma cholesterol, liver TG, and liver cholesterol. In terms of GM, astaxanthin reduced the abundance of *Proteobacteria* and the *Firmicutes*/*Bacteroidetes* ratio, while increasing the abundance of *Verrucomicrobia*, *Akkermansia*, and *Bacteroides*.

In the study reported by Ren et al. (2023) the anti‐tumor effect of astaxanthin on mice with HCC was assessed. Astaxanthin was associated with a higher quantity of tumor‐associated macrophages, infiltration of CD8+ T cells, Tumor Granzyme B, IL‐2, serum TNF‐α and interferon‐γ, genetic transcription of CXCL9, CXCL10, and CXCR3, and mRNA expression of cluster of differentiation 40. Astaxanthin regulated GM by elevating the abundance of *Akkermansia* and *Faecalibaculum* and attenuating the abundance of *Anaerotruncus*, *Gemella*, *Colidextribacter*, *Staphylococcus*, and *Enterococcus*. *Enterococcus*, *Anaerotruncus*, and *Colidextribacter* were negatively related to the tumor immune response and positively associated with tumor volume and mass.

**FIGURE 3:**
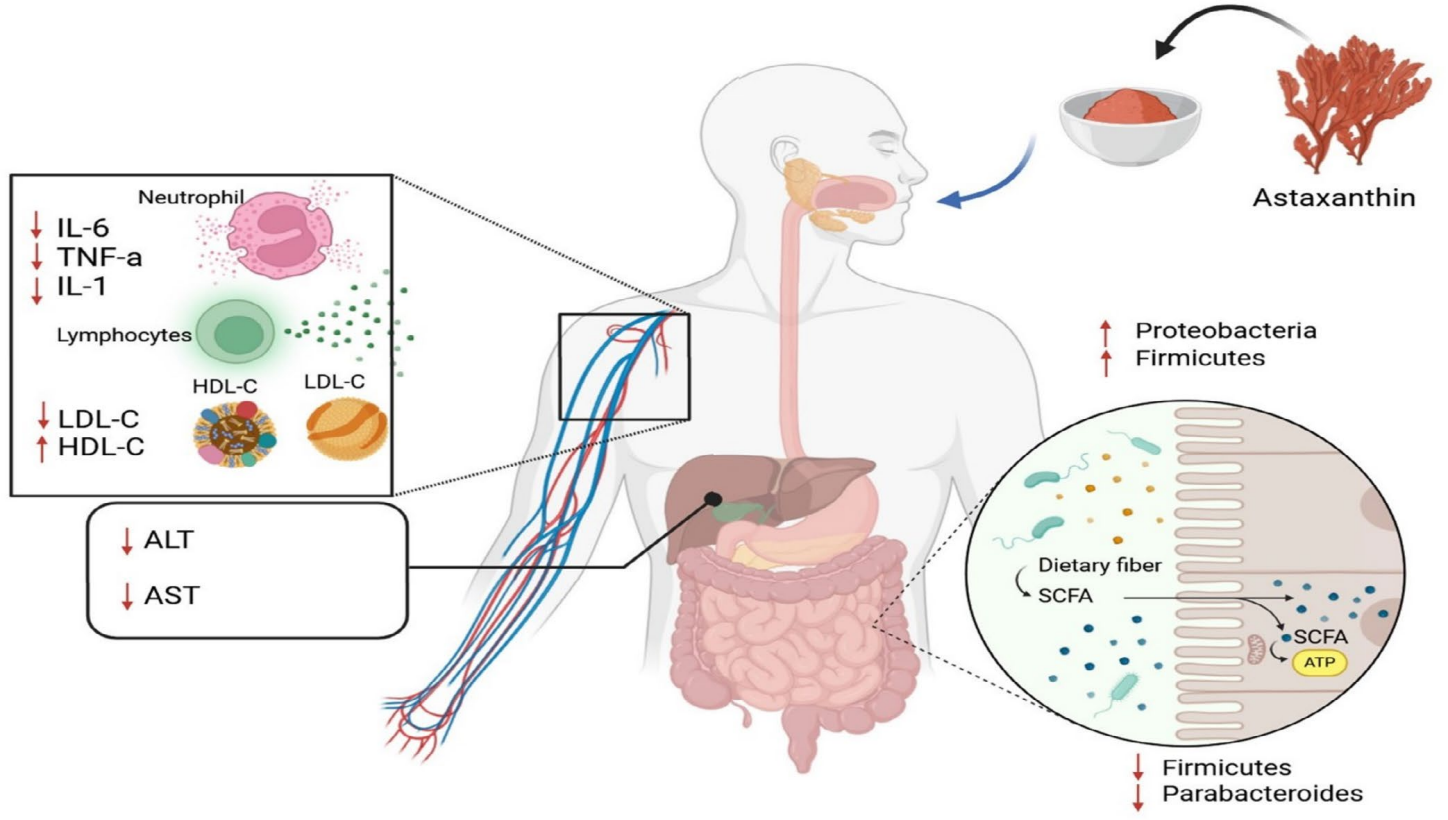
Astaxanthin reduces the expression of TNF‐α, IL‐6, and IL‐1 from immune cells and therefore the concentration of TNF‐α, IL‐6, and IL‐1 is reduced in serum; it also enhances the production of HDL from the liver, and reversely it reduces the production of LDL in serum. Astaxanthin modulated GM composition and attenuated the abundance of Bacteroidetes, Proteobacteria, Butyricimonas, Bilophila, and Parabacteroides while increasing the abundance of Verrucomicrobia and Akkermansia. IL‐1; Interleukin‐1, Interleukin 6, TNF‐α; Tumor necrosis factor, LDL; Low‐Density Lipoproteins, HDL; High‐Density Lipoprotein, SCFA; Short‐chain fatty acids, AST; Aspartate transaminase, ALT; Alanine Transaminase.

Liu et al. (2018) evaluated the effect of astaxanthin supplementation on ALD in mice. There was a significant association between astaxanthin intake and reduced liver fat accumulation, hepatic steatosis, LDL‐C, TG, AST, ALT, IL‐1, TNF‐α, IL‐6, and macrophage inflammatory protein −2 levels. Astaxanthin modulated GM and attenuated the abundance of *Bacteroidetes*, *Proteobacteria*, *Butyricimonas*, *Bilophila*, and *Parabacteroides* while elevating the abundance of *Verrucomicrobia* and *Akkermansia* Figure 3.

Wang et al. (2021) in another animal study examined astaxanthin's effect on obesity. They realized that astaxanthin intake reduced the food efficiency ratio, body weight gain, liver weight, perirenal and epididymal adipose tissue, serum TC, serum TG, serum LDL‐C, hepatic TG, and hepatic TC while elevating HDL‐C. Also, it increased PPARα, liver X receptor α, AMPK, adiponectin receptor 1, and PGC‐1α expression and reduced SREBP1c, FAS, and stearoyl CoA desaturase 1 (SCD‐1) expression. In terms of GM, astaxanthin attenuated the abundance of *Firmicutes*, *Proteobacteria*, and *Bifidobacterium*, whereas it elevated the abundance of *Bacteroidetes*, *Actinobacteria*, *S24‐7*, *Bacteroidales*, *Ruminococcaceae*, and *Akkermansia*.

Li et al. (2022) evaluated the effect of astaxanthin supplementation on HFD‐fed mice. They observed a significant correlation between astaxanthin consumption and lower daily food intake, body weight gain, lipid droplet vacuoles, liver index, epididymal fat index, IL‐17A expression, MDA, and serum and liver LDL‐C, TG, TC, AST, and ALT, whereas increased SOD, catalase, ferric reducing antioxidant power (FRAP), serum, and liver HDL‐C. Also, astaxanthin increased p‐AMPK, nuclear factor E2‐related protein 2, and heme oxygenase‐1 expression, whereas reduced SREBP‐1, ACC, and FAS expression. In terms of GM, it elevated the relative abundance of *Lactobacillus*, *Lactococcus*, *Dubosiella*, *[Eubacterium]_xylanophilum_group*, *Candidatus_Saccharimonas*, and *Faecalibacterium*. Furthermore, astaxanthin attenuated the abundance of *Desulfovibrionaceae* and *Rikenella*.

Wang et al. (2022) evaluated the effect of astaxanthin on HFD‐fed mice. They realized that astaxanthin reduced body weight gain, energy intake, liver weight, liver visual adipose tissue, liver TG and TC, serum TG, TC, and LDL‐C, the production of fatty droplets, and the apoptosis rate in the liver, serum AST, steatohepatitis score, and modulated the expression of genes related to lipid oxidation and bile acid metabolism. Furthermore, it increased antioxidant capacity, catalase, SOD, and GSH levels. Astaxanthin regulated GM by attenuating the abundance of *Firmicutes* and *Proteobacteria* and increasing the abundance of *Akkermansia* and *Parabacteroides*.

Zhang et al. (2024) assessed the effect of astaxanthin supplementation on liver injury of Kunming mice. There was a correlation between astaxanthin and lower serum TG, ALT, AST, LPS, and serum and hepatic MDA, TNF‐α, IL‐6, and IL‐1β. Furthermore, it increased hepatic levels of SOD, GSH‐PX, antioxidant capacity, fecal SCFAs, propionic acid, acetic acid, valeric acid, and isobutyric acid levels. In terms of GM, it reduced the abundance of *Firmicutes*, the *Firmicutes*/*Bacteroidetes* ratio, and *Lactobacillus*, whereas it increased the abundance of *Bacteroidetes*, *norank_f__norank_o_Clostridia_UCG‐014*, *norank_f__Lachnospiraceae*, *Prevotellaceae_UCG‐001*, *unclassified_f_Lachnospiraceae*, and *unclassified_f_Prevotellaceae*. *Bacteroidetes* were related to lower serum IL‐6, liver IL‐1β and higher liver antioxidant capacity. Firmicutes had a negative relationship with liver antioxidant capacity and GSH‐PX while had a positive correlation with serum TNF‐α, AST, and IL‐6 and hepatic MDA, IL‐1β, and IL‐6. The *Firmicutes*/*Bacteroidetes* ratio had a negative correlation with liver antioxidant capacity and a positive correlation with serum AST, TNF‐α, IL‐6, and hepatic IL‐1β and MDA. *Lactobacillus* had a negative association with liver SOD and a negative relationship with liver IL‐1β, IL‐6, MDA, serum TNF‐α, IL‐6, and AST. *Norank_f__Lachnospiraceae* showed a negative association with liver IL‐1β and a positive correlation with serum antioxidant capacity. *Unclassified_f__Lachnospiraceae* had a negative correlation with liver TNF‐α, MDA, and serum ALT. *Lachnospiraceae_UCG‐006* showed a positive association with serum antioxidant capacity, liver SOD, and GSH‐PX and a negative relationship with serum MDA and AST. *Norank_f__norank_o__Clostridia_UCG‐014* had a positive relationship with liver antioxidant capacity. *Unclassified_f__Prevotellaceae* indicated a positive association with liver SOD and a negative correlation with serum TNF‐α, ALT, liver TNF‐α, and MDA. *Streptococcus* had a positive association with serum SOD and a negative correlation with serum IL‐1β, TNF‐α, ALT, AST, liver TNF‐α, and MDA. *Prevotellaceae_UCG‐001* had a positive association with serum antioxidant capacity and SOD and a negative correlation with serum TNF‐α, IL‐1β, ALT, liver TNF‐α, and MDA. Also, *Rikenellaceae_RC9*_*gut_group* was negatively related to liver MDA and TNF‐α.

These studies showed that the intake of astaxanthin has positive properties on obesity and fatty liver by relieving inflammation and modulating GM and intestinal barrier function, but more studies are needed to confirm these results.

### 4.6 Lutein

Lutein is a type of organic pigment called a carotenoid with antioxidant effects, which is found in dark green leafy vegetables, egg yolk, animal fat, and the human eye retinal macula (Ahn and Kim 2021; Chung et al. 2004). Supplementation with lutein has been shown to effectively improve aberrant lipid metabolism, oxidative stress, inflammation, and gut barrier integrity (J.‐H. Kim et al. 2008; Nagira et al. 2006). The effect of lutein on fatty liver via modulating GM has been investigated recently.

Zhao et al. (2023) evaluated the effect of lutein supplementation on ALD Wistar rats. They found that lutein intake reduced Liver pathology scores, serum ALT, AST, TG, TNF‐α, IL‐1β, and LPS levels. Also, it modulated protein expression associated with oxidative stress and inflammatory pathways and increased hepatic GSH‐Px, catalase, SOD, and antioxidant capacity. Furthermore, lutein improved intestinal barrier integrity by elevating the expression of Claudin‐1, Occludin, and ZO‐1, ileum length, villus length, and villus length/crypt depth ratio while reducing FABP2 levels, intestinal villi breakage, and atrophy. In terms of GM, lutein elevated the abundance of *Eubacterium ruminantium* , *Faecalibacterium*, *Subdoligranulum*, and *Coprobacter*. *Faecalibacterium* had a positive association with SOD and a negative relationship with ALT and TNF‐α.

The previous study indicated that lutein has the potential to improve fatty liver via modulating GM composition. More animal studies and human clinical trials are needed to confirm these findings.

## 5 Conclusion

Our findings suggest that carotenoid supplementation, including lycopene, zeaxanthin, fucoxanthin, capsanthin, astaxanthin, and lutein, may have positive effects on obesity and fatty liver disease via modulating intestinal barrier function and gut microbiota. They indicated improvement in different factors related to obesity and fatty liver disease, such as energy expenditure, food intake, lipid profile, liver fat deposition, liver enzymes, inflammatory markers, glucose homeostasis, and bile acids. Although the results are promising, more comprehensive studies are needed to confirm and expand upon these findings and clarify the mechanisms behind the observed effects. However, further investigations are necessary to assess these supplements' long‐term safety and efficacy in managing obesity and fatty liver disease. The potential synergistic effects of combining carotenoids with other interventions for obesity and fatty liver disease should be explored in future studies.

## Author Contributions

**Dorna Hashemi:** conceptualization (lead), methodology (lead), writing – original draft (equal). **Mohammad Vahedi Fard:** data curation (equal), investigation (equal), writing – original draft (equal). **Kimia Mohammadhasani:** data curation (equal), investigation (equal), writing – original draft (equal). **Mehdi Barati:** visualization (lead), writing – review and editing (equal). **Elyas Nattagh‐Eshtivani:** project administration (lead), writing – review and editing (equal).

## Conflicts of Interest

The authors declare no conflicts of interest.

## Data Availability

The authors have nothing to report.
